# Advancements in Interventional Pulmonology: Harnessing Ultrasound Techniques for Precision Diagnosis and Treatment

**DOI:** 10.3390/diagnostics14151604

**Published:** 2024-07-25

**Authors:** Alireza Nathani, Sevak Keshishyan, Roy Joseph Cho

**Affiliations:** Division of Pulmonary, Allergy, Critical Care and Sleep Medicine, University of Minnesota, Minneapolis, MN 55455, USA; natha027@umn.edu (A.N.); keshi008@umn.edu (S.K.)

**Keywords:** bedside ultrasound, interventional pulmonology, transthoracic echocardiogram, transducer, ultrasound physics, endobronchial ultrasound

## Abstract

Medical ultrasound has emerged as an indispensable tool within interventional pulmonology, revolutionizing diagnostic and procedural practices through its non-invasive nature and real-time visualization capabilities. By harnessing the principles of sound waves and employing a variety of transducer types, ultrasound facilitates enhanced accuracy and safety in procedures such as transthoracic needle aspiration and pleural effusion drainage, consequently leading to improved patient outcomes. Understanding the fundamentals of ultrasound physics is paramount for clinicians, as it forms the basis for interpreting imaging results and optimizing interventions. Thoracic ultrasound plays a pivotal role in diagnosing conditions like pleural effusions and pneumothorax, while also optimizing procedures such as thoracentesis and biopsy by providing precise guidance. Advanced ultrasound techniques, including endobronchial ultrasound, has transformed the evaluation and biopsy of lymph nodes, bolstered by innovative features like elastography, which contribute to increased procedural efficacy and patient safety. Peripheral ultrasound techniques, notably radial endobronchial ultrasound (rEBUS), have become essential for assessing pulmonary nodules and evaluating airway structures, offering clinicians valuable insights into disease localization and severity. Neck ultrasound serves as a crucial tool in guiding supraclavicular lymph node biopsy and percutaneous dilatational tracheostomy procedures, ensuring safe placement and minimizing associated complications. Ultrasound technology is suited for further advancement through the integration of artificial intelligence, miniaturization, and the development of portable devices. These advancements hold the promise of not only improving diagnostic accuracy but also enhancing the accessibility of ultrasound imaging in diverse healthcare settings, ultimately expanding its utility and impact on patient care. Additionally, the integration of enhanced techniques such as contrast-enhanced ultrasound and 3D imaging is anticipated to revolutionize personalized medicine by providing clinicians with a more comprehensive understanding of anatomical structures and pathological processes. The transformative potential of medical ultrasound in interventional pulmonology extends beyond mere technological advancements; it represents a paradigm shift in healthcare delivery, empowering clinicians with unprecedented capabilities to diagnose and treat pulmonary conditions with precision and efficacy. By leveraging the latest innovations in ultrasound technology, clinicians can navigate complex anatomical structures with confidence, leading to more informed decision-making and ultimately improving patient outcomes. Moreover, the portability and versatility of modern ultrasound devices enable their deployment in various clinical settings, from traditional hospital environments to remote or resource-limited areas, thereby bridging gaps in healthcare access and equity.

## 1. Introduction

Interventional pulmonology represents a rapidly evolving subspecialty within pulmonary medicine, focusing on the diagnosis and management of complex pulmonary diseases through minimally invasive procedures. In recent years, medical ultrasound has emerged as a cornerstone technology in interventional pulmonology, offering unparalleled advantages in real-time imaging, procedural guidance, and patient management. This introduction explores the pivotal role of ultrasound in interventional pulmonology, highlighting its diverse applications, technological advancements, and clinical implications.

Ultrasound, utilizing high-frequency sound waves, has revolutionized medical imaging by providing detailed anatomical and functional information without ionizing radiation or invasive procedures. In interventional pulmonology, ultrasound serves as a versatile tool for guiding a wide range of procedures, including transthoracic needle aspiration, pleural interventions, endobronchial interventions, and percutaneous tracheostomy. Its ability to offer dynamic, real-time visualization of pulmonary structures and adjacent tissues enhances procedural accuracy, minimizes complications, and improves patient outcomes.

The use of ultrasound in interventional pulmonology encompasses various modalities, each tailored to specific clinical scenarios. Thoracic ultrasound enables the assessment of pleural abnormalities, pneumothorax, and diaphragmatic function with high sensitivity and specificity [1]. Endobronchial ultrasound facilitates the visualization of central airway structures and mediastinal lymph nodes, aiding in the diagnosis and staging of lung cancer and other mediastinal pathologies [2]. Peripheral lung ultrasound provides insights into peripheral pulmonary consolidations, interstitial lung disease, and pleural-based abnormalities, complementing traditional imaging modalities such as chest radiography and computed tomography (CT) [3]. Furthermore, ultrasound-guided percutaneous dilation tracheostomy enhances safety and accuracy by providing real-time visualization of anatomical structures, reducing the risk of complications [4].

The integration of advanced technologies, such as artificial intelligence (AI) algorithms, has further augmented the utility of ultrasound in interventional pulmonology. AI-driven ultrasound systems offer enhanced image resolution, automated analysis, and decision support, facilitating more accurate diagnoses and streamlined procedural workflows [5]. Moreover, the miniaturization of ultrasound probes and the development of portable and wearable ultrasound devices have expanded access to imaging in diverse clinical settings, including remote or resource-limited environments [6].

In addition to procedural guidance, ultrasound plays a crucial role in the assessment of treatment responses and post-procedural complications in interventional pulmonology. Serial ultrasound examinations enable clinicians to monitor changes in lesion size, vascularity, and tissue characteristics, guiding therapeutic decisions and optimizing patient care [7]. Furthermore, contrast-enhanced ultrasound techniques provide valuable insights into vascular perfusion and tissue microcirculation, enhancing the characterization of pulmonary lesions and guiding targeted interventions [8].

The widespread adoption of ultrasound in interventional pulmonology underscores the importance of comprehensive training and education for healthcare providers. Mastery of basic ultrasound physics, image acquisition techniques, and interpretation skills is essential for optimizing procedural outcomes and ensuring patient safety [9]. Furthermore, ongoing research and clinical trials are essential for advancing the field, exploring novel applications, and validating the efficacy of emerging technologies in interventional pulmonology.

Ultrasound has emerged as a fundamental technology in interventional pulmonology, offering unparalleled advantages in procedural guidance, diagnostic imaging, and patient management. Its diverse applications, technological advancements, and clinical implications underscore its pivotal role in transforming the field of pulmonary medicine. Through continued innovation, education, and research, ultrasound will continue to revolutionize interventional pulmonology, improving outcomes for patients with complex pulmonary diseases. This review aims to provide a comprehensive overview of the latest advancements in ultrasound application in interventional pulmonology by examining the current state of ultrasound techniques and future innovations in the field.

## 2. Relevant Sections

### 2.1. Ultrasound Physics

Ultrasound operates on the principles of sound wave physics to visualize internal structures of the body [10]. At its core, ultrasound employs high-frequency sound waves beyond the audible range of human hearing to create images of bodily tissues [11]. These waves are produced by a transducer, a crucial component that converts electrical energy into mechanical vibrations and vice versa. The piezoelectric effect serves as the backbone of ultrasound technology, particularly in the design and functionality of ultrasound transducers. This effect, first discovered by Jacques and Pierre Curie in 1880, describes the ability of certain materials to generate an electric charge in response to mechanical stress [12]. In ultrasound transducers, typically made of piezoelectric crystals like lead zirconate titanate (PZT), this effect is harnessed to both emit and receive ultrasonic waves. When an alternating electric current is applied to the piezoelectric crystal within the transducer, it undergoes mechanical deformation, creating ultrasonic waves that propagate into the body. These waves bounce off tissues and organs, producing echoes that are captured by the same transducer. Here, the piezoelectric crystal converts the returning mechanical vibrations back into electrical signals, which are then processed to generate images. The efficiency and precision of ultrasound imaging heavily rely on the performance of these piezoelectric ultrasound transducers [13]. Their ability to convert electrical energy into mechanical vibrations and vice versa with minimal energy loss enables high-resolution imaging. Moreover, advancements in piezoelectric materials and transducer design have led to improvements in imaging quality, depth penetration, and resolution over the years.

Grey scale imaging, or B (brightness) mode ultrasound, renders two-dimensional images in which organs and tissues of interest are depicted as different brightness. The formation of B-mode images is based upon the concept of the pulse echo principle. This states that the position of a target is based on the inferred time it takes an ultrasound wave to emit, travel to the target location, and return back to the transducer. Transducers come in various types, including linear, curved, phased array, and intracavitary, each suited to specific medical imaging needs. Characteristics of each transducer will be discussed below.

When ultrasound waves encounter different tissue densities, they undergo reflection, refraction, absorption, and scattering [14]. These interactions yield valuable information about the internal structures, helping to diagnose pathologies and monitor treatment responses. Moreover, the frequency of ultrasound waves influences image resolution and penetration depth. Higher frequencies, such as the linear probe (5–15 MHz), offer superior resolution but limited tissue penetration, making them suitable for superficial imaging, while lower frequencies (phased array probe 1–5 MHz), penetrate deeper into tissues but sacrifice resolution. As waves propagate, they experience attenuation, where energy dissipates due to absorption and scattering [15]. Understanding these propagation characteristics is vital for optimizing imaging parameters and ensuring diagnostic accuracy. To optimize image quality to help clinical decision-making, it is also important to recognize the cardinal transducer movements. These include sliding, tilting, rotating, and rocking [16]. From transducer technology to wave propagation and image formation, each aspect of ultrasound physics contributes to its efficacy in diagnosing diseases and guiding clinical interventions [17].

### 2.2. Neck Ultrasound

Neck ultrasound serves as a pivotal diagnostic tool, offering a non-invasive and dynamic assessment of various structures within the neck region [18]. This imaging modality enables clinicians to discern the intricate anatomy of the neck, facilitating the identification of crucial structures such as the thyroid gland, lymph nodes, and blood vessels. The thyroid gland, situated anteriorly, is meticulously evaluated for size, shape, and echogenicity, aiding in the diagnosis of thyroid disorders like nodules or inflammation. Additionally, lymph nodes scattered throughout the neck are scrutinized for size, shape, and echotexture alterations, assisting in the detection of pathologies such as metastatic disease or infection [19]. Furthermore, neck ultrasound proficiently delineates vascular structures, including the carotid arteries and jugular veins, offering insights into their patency, caliber, and presence of plaques or thrombi. This imaging modality’s real-time capability permits dynamic evaluation, allowing for the assessment of structures during swallowing or neck movement and enhancing diagnostic accuracy. Moreover, its non-ionizing nature renders it safe for repeated examinations, particularly beneficial in monitoring thyroid nodules or lymph node changes over time [20]. By harnessing high-frequency sound waves, neck ultrasound provides clinicians with detailed anatomical information crucial for formulating treatment plans and guiding interventional procedures. Ultimately, neck ultrasound stands as an indispensable tool in the armamentarium of clinicians, empowering them with the ability to diagnose a spectrum of neck pathologies while prioritizing patient safety and comfort swiftly and accurately.

**Figure 1:**
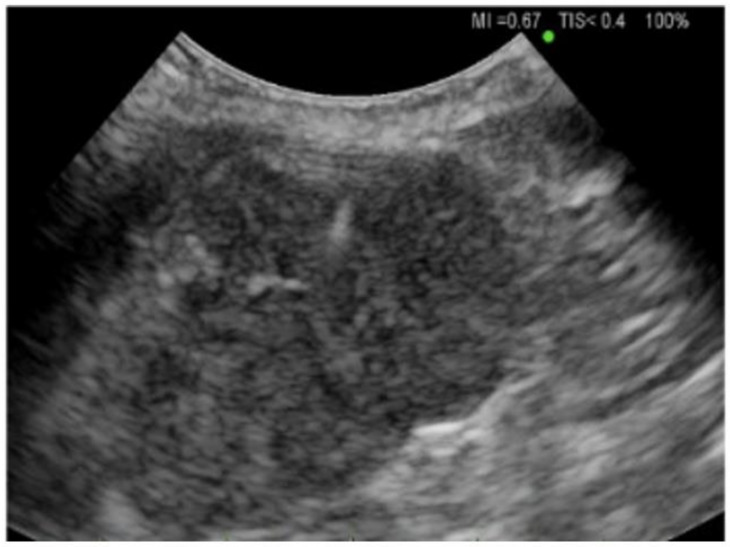
Ultrasound of a Supraclavicular Lymph Node. The lymph node is lobular, non-calcified and irregular which was consistent with metastatic lung cancer.

Tumor cells in a supraclavicular lymph node with non-small cell lung cancer indicates N3 and at least stage IIIB disease [21]. When indicated and performed over EBUS TBNA, this can safely be performed on patients requiring high oxygen requirements and with no sedation. In our practice, we generally locate a desired supraclavicular lymph node on computed tomography or positron emission tomography imaging and then confirm the presence and size of the lymph node using a high-frequency probe on the ultrasound (see Figure 1). Once the lymph node is identified along with other vasculature structures in the neck, the area is cleaned and draped in a sterile fashion. Local infiltration with lidocaine is administered to anesthetize the skin and tract. The head of the patient is turned to the contralateral side to help expose the supraclavicular area. Biopsy is performed in real-time under ultrasound guidance using a 21-gauge needle with a syringe attached to it to apply suction. Five to six agitations are performed. The ultrasound probe can either be held in the long or short axis depending on operator preference and whichever angle gives the best view of the needle piercing the lymph node. Cytopathology is usually at the bedside and can help with rapid onsite evaluation and adequacy of samples. There are several vasculature structures in the neck, and one must be aware of including the internal jugular vein, carotid artery, and the thyroid gland. Once completed, the puncture site is cleaned and dressed with a band-aid. Complications can include bleeding as well as pneumothorax. The cupola of the lung can be penetrated by the needle, and it is best practice to perform a lung ultrasound at the start and end of the procedure to assess for pneumothorax or changes in lung sliding.

**Figure 2:**
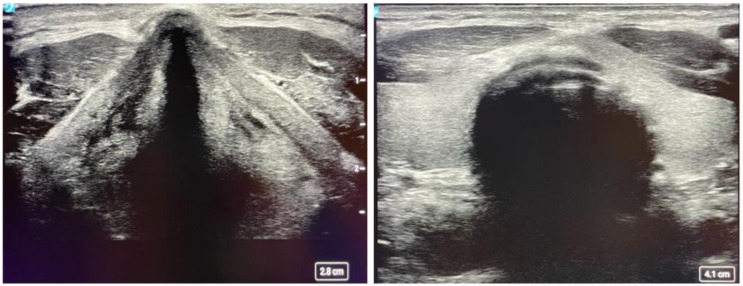
Ultrasound images for percutaneous dilational tracheostomy (PDT). Images are obtained from the level of the cricoid cartilage (**Left**) and thyroid (**Right**). The main reason to perform bedside ultrasound prior to PDT is to advance the needle by ultrasound approach and to avoid bleeding risk by identifying central blood vessels, including innominate vein and artery.

Ultrasound plays a pivotal role in the planning and execution of percutaneous dilational tracheostomy, offering crucial anatomical insights and guiding procedural accuracy. Through real-time visualization of the relevant structures such as the trachea, thyroid gland, blood vessels, and surrounding tissues, ultrasound facilitates precise localization of the ideal tracheal puncture site, minimizing the risk of complications such as vascular injury or posterior tracheal wall penetration (see Figure 2). Our approach is to use the high-frequency probe to assess anatomic structures in the neck before performing tracheostomy. We examine for aberrant blood vessels, thyroid isthmus, and central vasculature that would interfere with dilation and placement of tracheostomy. The ideal location for tracheostomy is between the first and second or the second and third tracheal ring. Palpation of landmarks can become particularly difficult in obese patients, and ultrasound can be immensely helpful in this population of patients. Additionally, ultrasound aids in assessing the depth and angulation of the trachea, optimizing the trajectory for safe tracheostomy tube placement. The utilization of ultrasound guidance enhances the safety and efficacy of PDT, making it an indispensable tool in the hands of skilled practitioners [22].

### 2.3. Thoracic Ultrasound

**Figure 3:**
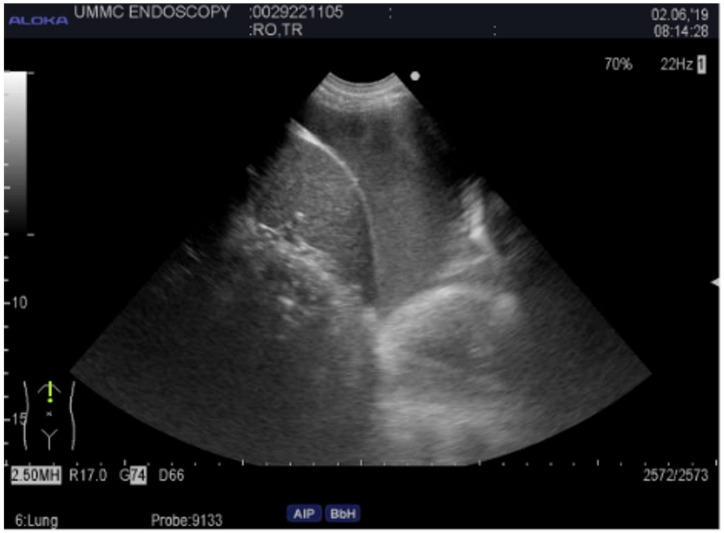
Ultrasound image of pleural effusion. Both thoracic and abdominal structures are seen, including the lung, diaphragm, and liver.

Pleural ultrasound has long been used to assist in the diagnosis and management of pleural effusions. A few key definitions are important to note. The echogenicity of a tissue refers to the ability to reflect or transmit ultrasound waves in the context of surrounding tissues [23]. In the pleural space, we frequently encounter anechoic fluid, which represents fluid that appears black on ultrasound. In contrast, hyperechoic refers to increasing density of sound waves compared to the surrounding structure, which can be seen in bone and fat calcifications (see Figure 3). Next, hypoechoic structures give off fewer echoes. They are darker than the surrounding structure. Examples include lymph nodes and tumors. The choice of probe used for thoracic ultrasound can vary depending on the clinical circumstance. For thoracentesis, we generally recommend using a phased array or curvilinear probe. The position of the patient is also vital in obtaining images. If a patient is supine, it is helpful to raise the ipsilateral arm and place it above the head to optimize exposure to the lateral thoracic cavity. Once the patient has been positioned appropriately, you can start examining their pleural space. It is not infrequent to encounter complex appearing pleural fluid. Sonographic features such as septations, loculations, and fibrinous strands have been associated with exudative pleural effusions [24]. Thoracentesis is a frequently performed procedure by pulmonologists to drain pleural fluid. One of the potential complications of this procedure can be a laceration of the intercostal artery. The optimal site is in the triangle of safety, which is the 4th to 6th intercostal space mid-axillary line; however, this is not always feasible due to pleural fluid location, body habitus, or patient comfort. Normally, the intercostal neurovascular bundle runs inferior to each rib; however, this can be highly variable. Thoracic ultrasound can be performed before a thoracentesis using a linear probe to examine where the intercostal arteries are located prior to the insertion of a catheter [25]. This technique can be particularly helpful in the older population whose neurovascular bundle starts to lose elasticity and does not follow typical anatomical rules. Not infrequently, the pocket of fluid in the pleural space is small, and insertion of a catheter requires exceptional skills or CT guidance. Using a sterile probe cover, ultrasound can aid in the direct visualization of guide wire placement into the pleural space when attempts without it have failed to access the space.

**Figure 4:**
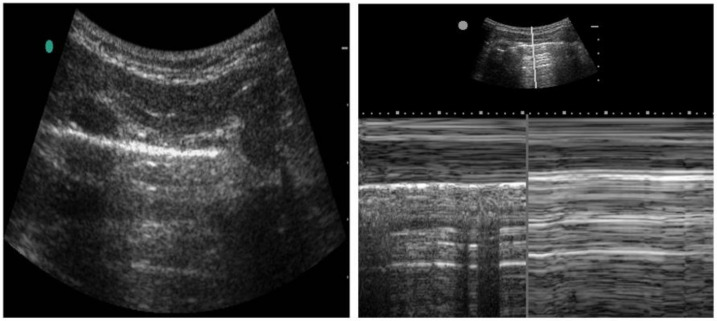
Ultrasound assessment for the pneumothorax. B-mode (**Left**) and M-mode (**Right**) technique for assessing pneumothorax. The B-mode demonstrates a normal visceral pleura lining (horizontal white line). The M-mode image demonstrates a normal pleura motion (**Left**) and abnormal pleural motion or ‘stratosphere sign’ (**Right**). The abnormal pleural motion is indicative of pneumothorax.

Assessment and treatment of pneumothorax is another common application that ultrasound has found its way to be helpful. Typically, the high-frequency probe is used to assess for pneumothorax, although the lower frequency probes can also be used. Lung sliding is a normal finding caused by the movement of visceral and parietal pleural against each other. Lack of lung sliding can be seen in conditions such as pneumothorax; however, it is not exclusive to this condition (see Figure 4). Lack of lung sliding also appears in any pathology that disrupts this process, including main stem intubation, severe acute respiratory distress syndrome, mucous plugging, and a history of pleurodesis. Lung pulse is another characteristic that can be seen in pneumothorax. Cardiac pulsation can be transmitted by pleura only if the pleural layers are adjacent, causing a small amount of motion in relation to the heartbeat [26]. The M mode takes the depth of your 2D B-mode image and plots it against time. For normal lungs, M-mode findings are described as a ‘sandy beach’, whereas a pneumothorax is described as a ‘stratosphere sign’ (Figure 4).

**Figure 5:**
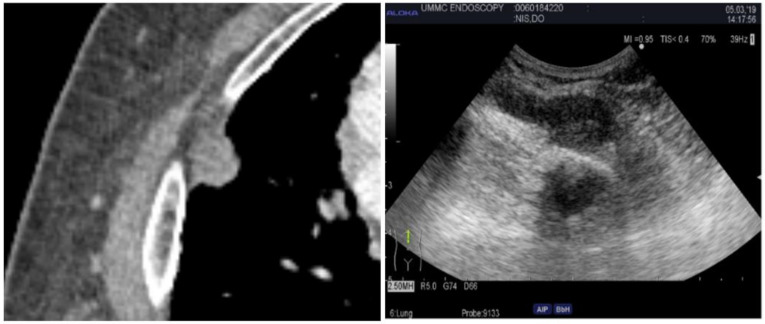
Computed tomography of the chest (**Left**) and corresponding percutaneous ultrasound (**Right**) of the nodule. The ultrasound image demonstrates ribs, pleura, and the nodule.

Lung masses can present in a variety of locations. For central masses, endobronchial ultrasound is the technique used to examine as they can be easily accessed through the tracheobronchial tree. For peripheral lung masses, there are different approaches to obtain tissue sample. One can navigate to the mass endobronchial and obtain samples through fine needle aspiration through a bronchoscope. This usually requires a navigation platform and general anesthesia. Another technique can be through interventional radiology and real-time CT guidance. An alternative to this is ultrasound-guided chest wall biopsy. The latter is safer with fewer complications and real-time visualization, no radiation exposure, and a diagnosis rate similar to that of CT, which the diagnostic accuracy is approximately 93.3% [27]. The ultrasound-guided technique is very similar to that of a supraclavicular lymph node biopsy. Depending on the size of the lesion, a high-frequency or phased array probe can be used to examine the lesion along the chest wall while the patient is laying supine (see Figure 5). Posterior lesions will be easier to examine while the patient is prone. Once the lesion is identified and the skin is anesthetized, ultrasound guidance can be used to agitate the lesion. The benefit of using ultrasound is that if at any point a pneumothorax is suspected or there is a change in vital signs, assessment of A and B lines can be performed rapidly to assess for pneumothorax. Pathology can also be at the bedside to assess for adequacy of samples.

### 2.4. Endobronchial Ultrasound

**Figure 6:**
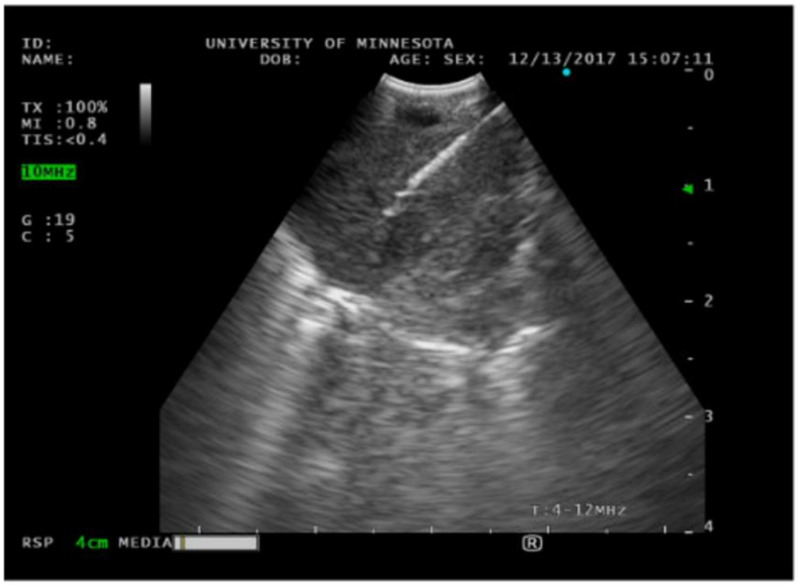
Endobronchial ultrasound image of a central lung mass. A needle is observed performing fine-needle aspiration.

Endobronchial ultrasound with transbronchial needle aspiration (EBUS TBNA) is considered one of the bread-and-butter techniques for pulmonologists and interventional pulmonologists. The most common indication for EBUS TBNA is lymphadenopathy, which can be attributed to benign or malignant conditions. Formerly, surgery was the only way to access mediastinal and hilar lymph nodes through mediastinoscopy. Over time and with advancement in technology, bronchoscopes with an ultrasound at the tip of the scope are now commonly utilized for this purpose. There are several companies that manufacture these bronchoscopes, including Olympus, Fuji, and Pentax. The benefit of ultrasound is multifold. The operator can evaluate the architecture of the lymph node, perfusion, resistance index, and elasticity [28]. Elastography is a non-invasive method where the relative stiffness of tissues is imaged as a color map or measured as a shear wave velocity. Most inflammatory processes do not change the architecture of lymph nodes; however, metastatic lymph nodes can cause hard infiltration of the lymph node. The Canada Lymph Node Score (CLNS) uses sonographic criteria during EBUS to predict the likelihood of malignant involvement [29]. The score gives a point to lymph nodes with features that suggest malignancy, including well-defined borders, absent central hilar structure, central necrosis present, and a small axis diameter less than or equal to ten millimeters. These are all findings that can be assessed with ultrasound during bronchoscopy (see Figure 6) and a combination of ultrasound features may accurately predict lymph node malignancy rather than a single feature. Moreover, turning on flow, one can assess for vasculature structures within the lymph node and areas to avoid biopsy. One of the challenges we face as interventional pulmonologists is confirming that our tools (i.e., the needle) are within the lesion. The great thing about EBUS is that we can see in real time our needle puncturing the lesion. Olympus recently introduced a new EBUS endoscope, the BF-UC190F, which has an outer diameter of 6.6 mm and more acute upward angulation of 160 degrees. This allows us to reach more distal targets in the lung, such as the right upper lobe, where prior to this device, this area was not commonly accessed with EBUS. Lung cancer staging is important as it defines prognosis, dictates therapy, and prevents unnecessary surgical interventions. Although PET scan is an excellent tool for diagnosis, its sensitivity and specify in lung cancer is 77 and 86%, respectively [30]. The sensitivity of EBUS TBNA is 92%. Endobronchial ultrasound of the esophagus, when combined with EBUS TBNA, gained an additional positive diagnostic yield of 7.1% [31]. Although EBUS TBNA has a high sensitivity for initial lung cancer staging, the sensitivity decreases after chemoradiation. Necrosis and fibrosis of the lymph nodes make fine needle aspiration more challenging to perform, and sensitivity drops to 76%. Negative endoscopic restaging exam should be, therefore, confirmed by surgical techniques [32]. In regard to lymphoma, which can also present with mediastinal and hilar lymphadenopathy, the sensitivity of EBUS to diagnose lymphoma is 57–61% [33]. Interestingly, the sensitivity is better at 88% for recurrent disease [34]. Granulomatous diseases such as sarcoidosis is another common reason for lymphadenopathy that pulmonologists see. The sensitivity of EBUS TBNA in this context is 83.3% compared with 60.9% using standard TBNA [35].

### 2.5. Peripheral Ultrasound

**Figure 7:**
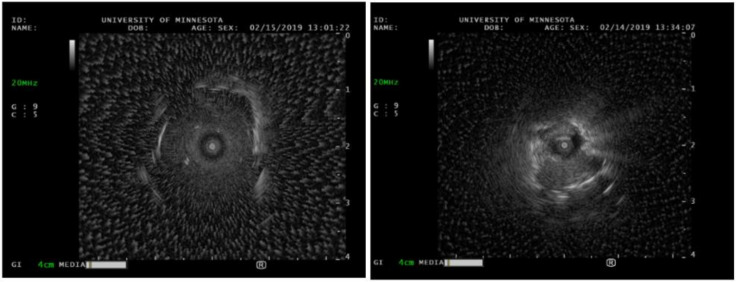
Radial EBUS ultrasound images of a peripheral nodule. The left image is consistent with the concentric signal, indicating that the radial EBUS probe is within the target. The right image is consistent with the eccentric signal, indicating that the radial EBUS probe is tangential to the target.

Peripheral bronchoscopic technology has exploded over the past decade with robotic platforms to assist with diagnosis; however, radial endobronchial ultrasound is still one of the core pieces of technology that is almost universally used by interventional pulmonologists (see Figure 7). A radial probe is advanced through the working channel of a bronchoscope or robotic arm. The ultrasound waves are translated into a 360-degree grey scale image of the lung parenchyma or target lesion [36]. Most commonly, it is used to evaluate pulmonary nodules. In a randomized trial, the diagnostic accuracy of radial probe EBUS-guided transbronchial biopsy was found to be similar to that of CT-guided biopsy, 87.5 and 93.3%, respectively [37]. Radial EBUS views of peripheral lesions are typically characterized in two ways: concentric and eccentric. Concentric is defined as the radial probe appearing to be completely surrounded by the lesion, whereas eccentric is when the probe is biased towards one side of the lesion without tissue completely surrounding it [38]. A concentric view has been shown to have a superior diagnostic yield when compared with an eccentric view. Robotic platforms, such as Intuitive Ion, allows integration of radial EBUS to allow for optimization of biopsy. Although radial EBUS does not allow for real-time sampling, there have been developments of ultrasound guidance systems using radial EBUS that facilitate pulmonary needle biopsies with simultaneous real-time visualization [39]. Although there are case reports demonstrating its effectiveness, robust studies have not yet been published. Moreover, radial EBUS can also be used in the setting of subglottic or tracheal stenosis. Tracheal stenosis is a form of central airway obstruction, often misdiagnosed as vocal cord dysfunction or asthma. There are a variety of etiologies for tracheal stenosis, including gastric reflux disease, prior history of endotracheal intubation, inflammatory conditions such as granulomatosis with polyangiitis, or idiopathic. While performing intra-operative radial EBUS of the affected area, the bronchoscopist can assess for cartilaginous integrity, as this is important in defining the complexity of the stenosis and potential treatments [40]. For example, balloon dilation of a stenotic segment does not work well with cartilaginous destruction and may require other forms of therapy.

## 3. Future Directions

The future of ultrasound in interventional pulmonology holds immense promise, driven by ongoing technological advancements and innovative research endeavors. One significant direction involves the integration of artificial intelligence (AI) algorithms into ultrasound systems, enabling automated image analysis, lesion characterization, and procedural guidance [41]. AI-driven ultrasound holds the potential to enhance diagnostic accuracy, streamline procedural workflows, and improve patient outcomes by providing real-time decision support to clinicians. Furthermore, the development of novel ultrasound contrast agents and molecular imaging probes promises to expand the diagnostic capabilities of ultrasound in interventional pulmonology [42]. These agents enable targeted imaging of specific molecular markers associated with pulmonary diseases, facilitating early detection, precise localization, and personalized treatment strategies.

Miniaturization of ultrasound devices and the advent of handheld, portable systems are poised to transform the delivery of interventional pulmonology services [43]. Portable ultrasound devices offer flexibility, accessibility, and point-of-care capabilities, enabling clinicians to perform procedures in diverse clinical settings, including outpatient clinics, emergency departments, and remote locations. This expansion of access to ultrasound technology has the potential to democratize healthcare, particularly in underserved communities with limited access to traditional imaging modalities.

Advancements in three-dimensional (3D) ultrasound imaging techniques hold promise for enhancing procedural planning, intraoperative navigation, and post-procedural assessment in interventional pulmonology [44]. 3D ultrasound enables volumetric reconstructions of pulmonary structures, providing clinicians with comprehensive anatomical information and improving procedural precision. Furthermore, real-time fusion imaging, which combines ultrasound with other imaging modalities such as computed tomography (CT) or magnetic resonance imaging (MRI), offers synergistic advantages for guiding complex interventions and improving localization accuracy [45].

Another future direction involves the development of enhanced ultrasound probes with advanced features such as high-frequency imaging, extended penetration depth, and improved spatial resolution [46]. These next-generation probes enable visualization of fine anatomical details, characterization of small pulmonary lesions, and precise needle guidance during interventions. Furthermore, the incorporation of innovative imaging modalities, such as elastography and photoacoustic imaging, into ultrasound systems offers additional insights into tissue biomechanics and molecular composition, augmenting diagnostic capabilities in interventional pulmonology [47].

Ongoing research efforts are focused on refining ultrasound-guided therapeutic interventions, such as targeted drug delivery, thermal ablation, and gene therapy, for the treatment of pulmonary diseases [48]. Ultrasound-guided interventions offer precise targeting, minimal invasiveness, and real-time monitoring, thereby minimizing collateral damage to healthy tissues and improving therapeutic efficacy. These emerging therapeutic approaches have the potential to revolutionize the management of pulmonary diseases, offering personalized treatment options and improved outcomes for patients.

Digital ultrasound transducers represent a significant advancement in medical imaging technology, offering improved image quality, versatility, and diagnostic capabilities [49]. Unlike traditional analog transducers, digital transducers incorporate digital signal processing (DSP) capabilities directly within the transducer housing. This integration allows for the conversion of analog ultrasound signals into digital data within the transducer itself, enabling real-time processing and analysis of the acquired images. Digital transducers utilize advanced algorithms to enhance image clarity, reduce noise, and optimize image resolution, resulting in sharper and more detailed diagnostic images. Additionally, digital technology enables the implementation of advanced imaging modalities such as harmonic imaging, speckle reduction, and elastography, which further enhance diagnostic accuracy and provide valuable clinical information.

The transition to digital ultrasound transducers has been facilitated by advancements in microelectronics, allowing for the integration of high-speed analog-to-digital converters (ADCs) and powerful signal processing algorithms within compact transducer designs. These transducers offer improved sensitivity, dynamic range, and spatial resolution compared to their analog counterparts, enabling clinicians to visualize subtle anatomical structures and pathological findings with greater clarity and confidence. Furthermore, digital transducers support the seamless integration of imaging data into electronic medical records (EMRs) and picture archiving and communication systems (PACS), facilitating efficient image storage, retrieval, and sharing across healthcare networks.

Several studies have demonstrated the clinical benefits of digital ultrasound transducers in various medical specialties, including obstetrics, cardiology, and musculoskeletal imaging. For example, research has shown that digital transducers can improve the detection of fetal anomalies, enhance visualization of cardiac structures, and provide more accurate assessments of tendon and ligament injuries. Moreover, digital transducers have been shown to increase workflow efficiency, reduce examination times, and enhance patient throughput in busy clinical settings [50,51]. As digital ultrasound technology continues to evolve, ongoing research and development efforts aim to further improve image quality, enhance diagnostic capabilities, and expand the range of clinical applications.

## 4. Conclusions

In conclusion, the future directions of ultrasound in interventional pulmonology are marked by exciting advancements in technology, imaging techniques, and therapeutic applications. From AI-driven automation and portable devices to advanced imaging modalities and targeted therapies, ultrasound continues to evolve as a pivotal tool in the diagnosis, treatment, and management of pulmonary diseases. By embracing these innovations and fostering interdisciplinary collaboration, clinicians and researchers can harness the full potential of ultrasound to improve patient care and advance the field of interventional pulmonology.
